# Computational Design of Inhibitors Targeting the Catalytic β Subunit of *Escherichia coli* F_O_F_1_-ATP Synthase

**DOI:** 10.3390/antibiotics11050557

**Published:** 2022-04-22

**Authors:** Luis Pablo Avila-Barrientos, Luis Fernando Cofas-Vargas, Guillermin Agüero-Chapin, Enrique Hernández-García, Sergio Ruiz-Carmona, Norma A. Valdez-Cruz, Mauricio Trujillo-Roldán, Joachim Weber, Yasser B. Ruiz-Blanco, Xavier Barril, Enrique García-Hernández

**Affiliations:** 1 Instituto de Química, Universidad Nacional Autónoma de México, Ciudad Universitaria, Ciudad de México 04510, Mexico; lpablo@comunidad.unam.mx (L.P.A.-B.); fcofas@comunidad.unam.mx (L.F.C.-V.); enrique.hernandez@iquimica.unam.mx (E.H.-G.); 2 CIMAR/CIIMAR, Centro Interdisciplinar de Investigação Marinha e Ambiental, Universidade do Porto, Terminal de Cruzeiros do Porto de Leixões, Av. General Norton de Matos, s/n, 4450-208 Porto, Portugal; gchapin@ciimar.up.pt; 3 Departamento de Biologia, Faculdade de Ciências, Universidade do Porto, Rua do Campo Alegre, 4169-007 Porto, Portugal; 4 Institut de Biomedicina de la Universitat de Barcelona (IBUB) and Facultat de Farmàcia, Universitat de Barcelona, Av. Joan XXIII s/n, 08028 Barcelona, Spain; sruizcarmona@gmail.com; 5 Programa de Investigación de Producción de Biomoléculas, Departamento de Biología Molecular y Biotecnología, Instituto de Investigaciones Biomédicas, Universidad Nacional Autónoma de México, Cd. Universitaria, Ciudad de México 04510, Mexico; adri@biomedicas.unam.mx (N.A.V.-C.); maurotru@biomedicas.unam.mx (M.T.-R.); 6 Department of Chemistry and Biochemistry, Texas Tech University, Lubbock, TX 79409, USA; joachim.weber@ttu.edu; 7 Center of Medical Biotechnology, Faculty of Biology, University of Duisburg-Essen, 45127 Essen, Germany; 8 Departament de Farmacia i Tecnología Farmacèutica, i Fisicoquímica, Institut de Biomedicina (IBUB), Universitat de Barcelona, Av. Joan XXIII, 27-31, 08028 Barcelona, Spain; xbarril@ub.edu; 9 Catalan Institution for Research and Advanced Studies (ICREA), 08010 Barcelona, Spain

**Keywords:** F_O_F_1_-ATP synthase, allosteric inhibition, structure-based drug design, evolutionary and PPI algorithms, peptide design

## Abstract

With the uncontrolled growth of multidrug-resistant bacteria, there is an urgent need to search for new therapeutic targets, to develop drugs with novel modes of bactericidal action. FoF1-ATP synthase plays a crucial role in bacterial bioenergetic processes, and it has emerged as an attractive antimicrobial target, validated by the pharmaceutical approval of an inhibitor to treat multidrug-resistant tuberculosis. In this work, we aimed to design, through two types of in silico strategies, new allosteric inhibitors of the ATP synthase, by targeting the catalytic β subunit, a centerpiece in communication between rotor subunits and catalytic sites, to drive the rotary mechanism. As a model system, we used the F1 sector of Escherichia coli, a bacterium included in the priority list of multidrug-resistant pathogens. Drug-like molecules and an IF1-derived peptide, designed through molecular dynamics simulations and sequence mining approaches, respectively, exhibited in vitro micromolar inhibitor potency against F1. An analysis of bacterial and Mammalia sequences of the key structural helix-turn-turn motif of the C-terminal domain of the β subunit revealed highly and moderately conserved positions that could be exploited for the development of new species-specific allosteric inhibitors. To our knowledge, these inhibitors are the first binders computationally designed against the catalytic subunit of FOF1-ATP synthase.

## 1. Introduction

At the end of the last century, there were already alarming signs of a growing health crisis because of the emergence of antimicrobial resistance (AMR) [1], which, if left unattended, would cause worldwide mass fatalities and colossal financial burden [2,3]. As it was feared, the decline in investment in the development of novel antibiotics has aggravated this crisis, reflected in the decrease in newly approved antibiotics, although a slight change in this trend was recently reported [4]. AMR microorganisms have developed effective antibiotic evasion mechanisms [5]. The need to circumvent those mechanisms prompts the search for novel pharmacological targets [4]. Bacterial bioenergetic pathways have recently unveiled a new Achilles heel to combat AMR [6], as evidenced by bedaquiline, the first approved anti-tuberculosis drug in 40 years, which targets *Mycobacterium tuberculosis* ATP synthase [7]. Furthermore, mounting evidence supports that blocking the catalytic activity of this enzyme sensitizes AMR facultative anaerobic microorganisms (*v. gr*., *Staphylococcus aureus* and *Escherichia coli*) to the action of other antimicrobial agents [8,9,10]. Therefore, ATP synthase appears as a momentous pharmacological target to broaden the battlefront against the pathogens of major concern.

**Figure 1:**
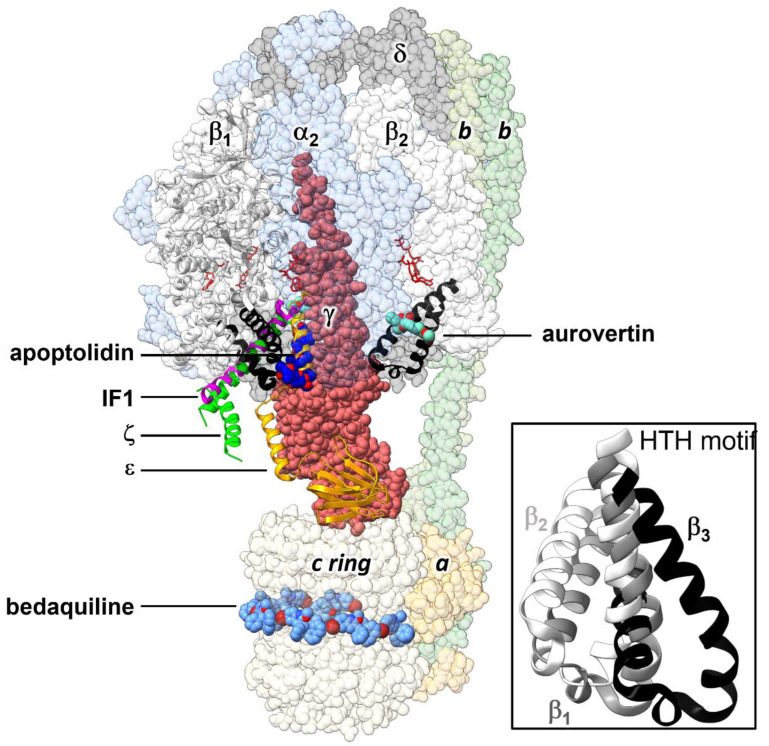
Schematic representation of the 3D structure of *E. coli* F_O_F_1_-ATP synthase, showing the binding sites of allosteric inhibitors that interact with the C-terminal domain of the β subunit. Inhibitors whose 3D structure in complex with the enzyme has been experimentally solved were docked by alignment on the cryoEM structure of the *E. coli* enzyme in an inhibited conformation by the ε subunit (PDB ID 6oqr [16]). HTH motifs of β_1_ and β_2_ are in black ribbons. The F_1_ and F_O_ subcomplexes are composed of the α_3_β_3_γδε and *ab*_2_*c*_10_ subunits, respectively. The endogenous inhibitory ε (PDB ID 6oqr [16]), ζ (PDB ID 5dn6 [23]) and IF1 (PDB ID 1ohh [24]) subunits are shown in ribbons. The exogenous inhibitors aurovertin B (PDB ID 1cow [25]) and the glycomacrolide apoptolidin A (PDB ID 7md3 [26]) are shown in spheres. Here, also shown is the position of the anti-tuberculosis drug bedaquiline (PDB ID 7jg8 [27]), which occupies sites equivalent to those of oligomycin in the *c* ring. Nucleotides are shown in sticks. **Inset:** Alignment of the HTH motifs of the three β subunits observed in the self-inhibited structure of *EcF_1_* [16,22]. In this conformation, the ε subunit hampers the closing of one β subunit, adopting a half-closed conformation (β_1_). A β_1_-like conformation is also observed in the complexes with the glycomacrolides apoptolidin A and ammocidin A [26].

ATP synthase is a sophisticated molecular motor, with an efficiency of ~100% [11], made up of two functionally coupled subcomplexes: a membrane embedded proton channel, F_O_, and a soluble catalytic subcomplex, F_1_. Together, F_O_ and F_1_ harvest electrochemical gradient potential energy to produce rotational energy that is eventually converted into chemical energy as a phosphodiester bond [12,13]. The enzyme also catalyzes, with high efficiency, the hydrolysis of ATP, being able to restore the proton gradient under physiological demand (v.gr., to generate membrane potential in bacteria under anaerobic conditions) [14]. The minimal architecture of this enzyme is found in bacteria (Figure 1), composed of eight types of subunits, with F_O_:*ab*_2_*c*_10-17_ and F_1_:α_3_β_3_γδε stoichiometries [12,15]. Proton translocation (or sodium ions, in some species) drives the rotation of the transmembrane ring of *c* subunits relative to the *a* subunit. This rotation drives the torque of the asymmetric γ subunit, which is partially embedded in the catalytic α_3_β_3_ ring. α_3_β_3_ is stabilized against rotation by the stator stalk, composed of δ, *b*, and *a* subunits in bacteria [16], and by a larger number of different subunits in mitochondria [17]. ATP synthase operates under a mechanism dubbed as the binding change mechanism [18]. Each of the three catalytic sites, composed mainly of residues of the β subunit and some of the α subunit, transits through three alternating affinity states, corresponding to three different conformational states. According to the nucleotide occupancy exhibited in the first experimental F_1_ structure from *Bos taurus* (BsF_1_) [19], these states are usually termed as β_E_ (empty binding site), β_TP_ (ATP bound), and β_DP_ (ADP bound). When the enzyme acts as a hydrolase, in an alternate progression, each β subunit goes in the order β_E_→β_TP_→β_DP,_ as the catalytic cycle progresses. The conformational changes in the β subunits are coupled to the formation and breakdown of contacts with the asymmetric α-helices of the γ subunit and the adjacent α subunits. In this rotary mechanism, the helix-turn-helix (HTH) motif of the β-subunit C-terminal domain (βCterm) plays a central role in the communication with the other subunits and has been described as a pushrod, pushed by the γ subunit (or which sets the γ subunit in motion in the hydrolysis direction) [20,21]. βCterm is in an open conformation in β_E_, with minimal intercatenary interactions. After 120° rotation of the γ subunit, driven by ATP binding, βCterm transits into a closed conformation in β_TP_, contacting to the γ subunit and one of the adjacent α subunits (α_TP_). A further 120° γ-subunit rotation leads to β_DP_, a conformation very similar to β_TP,_ except for tighter packing of its βCterm against the γ subunit and the adjacent α subunit (α_DP_). ATP hydrolysis and ADP release occur within 0°→~90° and ~90°→120° rotation substeps in the β_TP_→β_DP_ and β_DP_→β_E_ transitions, respectively [13]. In the self-inhibited conformation of the *Escherichia coli’s* F_1_ (EcF_1_), the β subunits β_2_ and β_3_ exhibit β_E_- and β_TP_-like conformations, respectively, while β_1_ adopts a half-closed conformation because of a steric hindrance of the C-terminal domain of the ε subunit in an extended conformation (Figure 1) [16,22].

A wealth of exogenous and endogenous ATP synthase inhibitors has been described [28,29]. Structural studies have identified several binding sites along the ATP synthase architecture for these inhibitors, revealing the existence of a diversity of allosteric mechanisms to inhibit the enzyme (Figure 1). Many of these inhibitors bind to sites involving βCterm. The eukaryotic inhibitor IF1 [30], and the prokaryotic ε and ζ subunits insert an α-helix motif into a pocket formed by the α_3_β_3_ ring and the γ subunit, near the C-terminal domains of α_DP_ and β_DP_, thus, stopping rotational catalysis and preventing wasteful ATP consumption [31,32,33]. Recently, the binding site of a family of glycomacrolide inhibitors was identified, in a region involving the HTH motif of a β_1_-like subunit [26]. In addition, aurovertins, antibiotics produced by the fungus *Calcarisporium arbuscula,* target equivalent sites in the bovine β_E_ and β_TP_ subunits between βCterm and the nucleotide binding domain [25]. Taken together, the existence of these non-orthosteric inhibitors indicates that βCterm is a suitable target for the development of allosteric pharmacological modulators. Because of a less stringent evolutionary pressure, allosteric sites tend to be less conserved than catalytic sites [34,35,36]. This aspect is relevant to design specific ATP synthase inhibitors, since some regions of the active site of this enzyme are highly conserved across P-loop NTPases [37,38]. Furthermore, allosteric inhibitors do not compete with the substrate, so they do not require reaching an extremely high binding potency to exert an effective pharmacological action [39]. Target-based allosteric inhibitor design on ATP synthase has been limited. GaMF1 [40] and epigallocatechin gallate [41] are a notable exception. These compounds, which inhibit mycobacterial ATP synthase by binding to γ and ε subunits, respectively, have been obtained through pharmacophoric-restraints filtered docking studies.

Given the crucial role played by the βCterm in driving the rotational mechanism of F_O_F_1_-ATP synthase, in this work, we set out to design, through two types of in silico strategies, new allosteric inhibitors, by targeting the HTH motif of the *Escherichia coli* F_1_ (EcF_1_), a bacterium included in the ESKAPEE (*Enterococcus faecium*, *Staphylococcus aureus*, *Klebsiella pneumoniae*, *Acinetobacter baumannii*, *Pseudomonas aeruginosa*, *Enterobacter spp*, and *Escherichia coli*) list of the most threatening AMR microbes [42]. The underlying idea was that the engineered binders, by interfering with the conformational transitions of this motif, will exert an allosteric effect, leading to the blocking of the enzyme’s rotation. On the one hand, through a molecular dynamics (MD) simulation approach with solvent mixtures (MDmix), we identified solvent sites (SS) on the HTH motif that helped guide the high-throughput virtual screening (HTVS) of drug-like molecules [43,44]. The best hits were further filtered using the dynamic undocking method (DUck), an orthogonal technique that evaluates the work developed upon pulling the ligand out from the binding site [45]. Using a new in silico strategy, guided by evolutionary and machine learning-based methods [46], we derived peptides from IF1, based on the observation that the residues that bind this inhibitor in BsF_1_ are highly conserved in EcF_1_. Both approaches led to the identification of drug-like and peptide hits that inhibited the hydrolytic activity of EcF_1_ with micromolar potency. Remarkably, given the different nature of the identified hits and the distinct modeling approaches, we proved the feasibility of the in silico design of ATPase inhibitors, targeting the catalytic subunit. These molecules could serve as leading scaffolds for the development of novel drugs to combat AMR bacterial strains.

## 2. Results

### 2.1. Structure-Based Design of Small Organic Inhibitors

**Figure 2:**
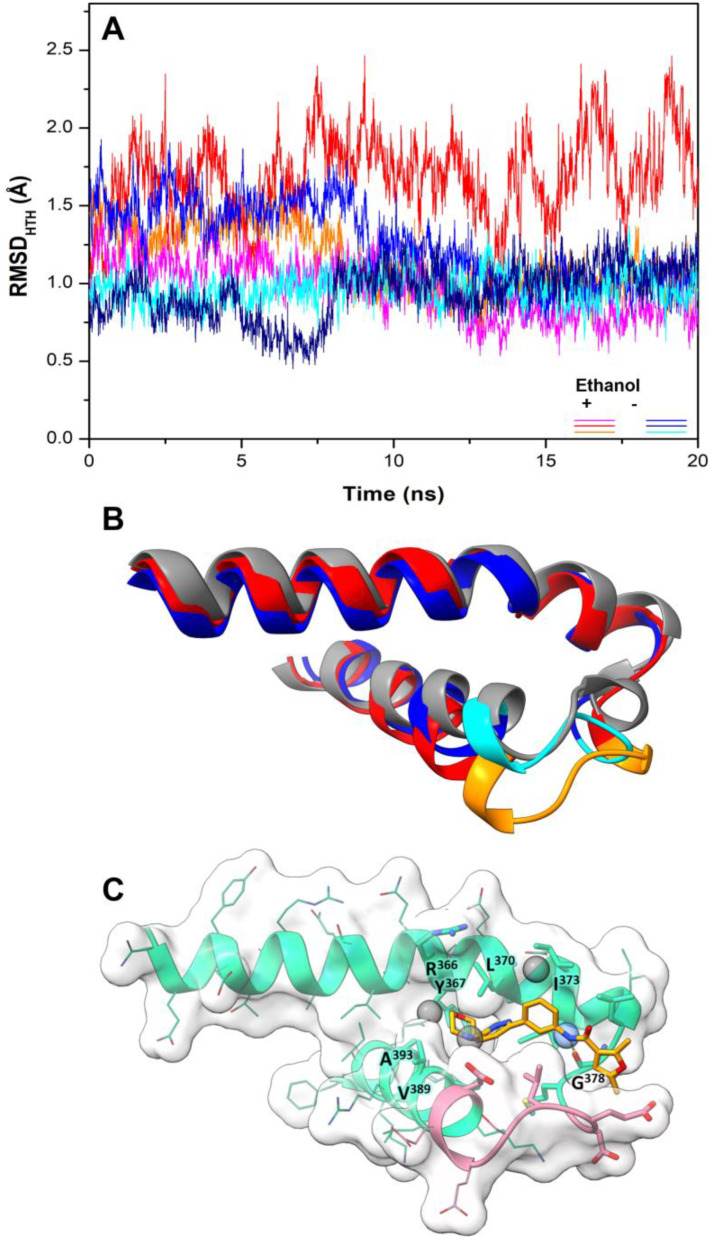
HTH dynamics and solvent sites determined from MD simulations. (**A**) RMSD was calculated using backbone atoms of the β_1_ HTH motif. Values from three MD simulations in pure water (blue colors) and three in water-ethanol mixture (red colors) are shown. (**B**) Average conformations of the HTH motif calculated over the last 10 ns of simulation. The open conformation obtained in one trajectory in a solvent mixture is shown in orange, while a closed conformation from one of the replicas in pure water is shown in blue. The DELSEED motif is in lighter color. The starting crystal conformation is in gray (PDB ID 3oaa, [22]). (**C**) Solvent sites for ethanol’s methyl (gray spheres) and hydroxyl (blue sphere) groups determined from one trajectory in ethanol/water mixture. One of the ligands (Compd-5) obtained in this work by HTVS (vide infra) is shown in sticks, forming a hydrogen bond with the carbonyl group of G^378^. The DELSEED motif is in pink.

For the design of drug-like molecules, based on the structure of ATP synthase, we performed MD simulations using the crystallographic structure of EcF_1,_ self-inhibited by the ε subunit and MgADP (Figure 1, PDB ID 3oaa), a distinctive conformational state of the enzyme in bacteria and chloroplasts [16,22]. In this structure, β_2_ and β_3_ show conformations very close to bovine β_E_ and β_TP_, respectively. In contrast, β_1_ adopts an intermediate conformation, since the ε subunit impedes the total closure of βCterm (Figure 1 inset), a mechanism hypothesized to prevent the enzyme from falling to a low-energy state, inhibited by MgADP [47]. To sample the conformational space neighboring, the crystal structure of Ec*F_1_*, unrestrained 20-ns simulations were performed. Judging by the time evolution of the RMSD (Figure 2A), the HTH of β_1_ converged to the same conformation in all solvent and pure water replicas, except for one trajectory in ethanol, in which a slightly more open conformation of the DELSEED motif was observed (Figure 2B). The analysis of this trajectory revealed the well-defined presence (ΔG_SS_ ≤ −1 kcal/mol) of a solvent site cluster, in an area close to the DELSEED region and intermediate between the two HTH helices. The cluster was composed of a site for the hydroxyl’s oxygen (SS_OH_) and three sites for the methyl carbon (SS_CT_) of ethanol (Figure 2C). The carbonyl group of G^378^ stabilized the unique SS_OH_, while the side chains of R^366^, Y^367^, I^373^, V^389^, and A^393^ were involved in the stabilization of the SS_CT_. Consistently, the four solvent sites were closely reproduced in three MD replicas, in which harmonic constraints (*k* = 0.5 kcal/molÅ^2^) were imposed on the heavy atoms of the protein to keep the HTH in open conformation (data not shown). Therefore, this open conformation of HTH, which was also significantly, albeit to a lesser degree, populated in pure water replicas, was apparently stabilized by the organic solvent.

The four solvent sites observed in β_1_ were used as pharmacophoric restraints to guide HTVS of ~8 × 10^6^ molecules from eight different commercial chemical libraries, using the rDock software [44]. After removing molecules with very similar chemical structures by visual inspection, the 100 top-ranked molecules, ordered according to the docking score, were additionally filtered using steering dynamics, with the DUck method [45]. The work required (W_QB_) to move away the hydrogen donor atom of the docked molecule to 5 Å (quasi-bound state) from the carbonyl group of G^378^ was determined, discarding those molecules with W_QB_ < 6 kcal/mol. As a result, 27 potential ligands were selected and purchased (Table S1). The inhibitory potency of these compounds was assayed against purified EcF_1,_ using the malachite green method. As shown in Table 1, five compounds displayed significant inhibition of ATPase activity at 100 µM of inhibitor concentration. Measurements performed using the NADH-linked ATP regeneration system yielded similar inhibition values. According to the Chemaxon solubility predictor server [48], the five compounds are soluble in the micromolar range (Table 1). It is also worth mentioning that, according to the MOE software, the five hit molecules are not pan-assay interference compounds (PAINS). Only compound 18 and 26 contained a PAINS warning.

**Figure 3:**
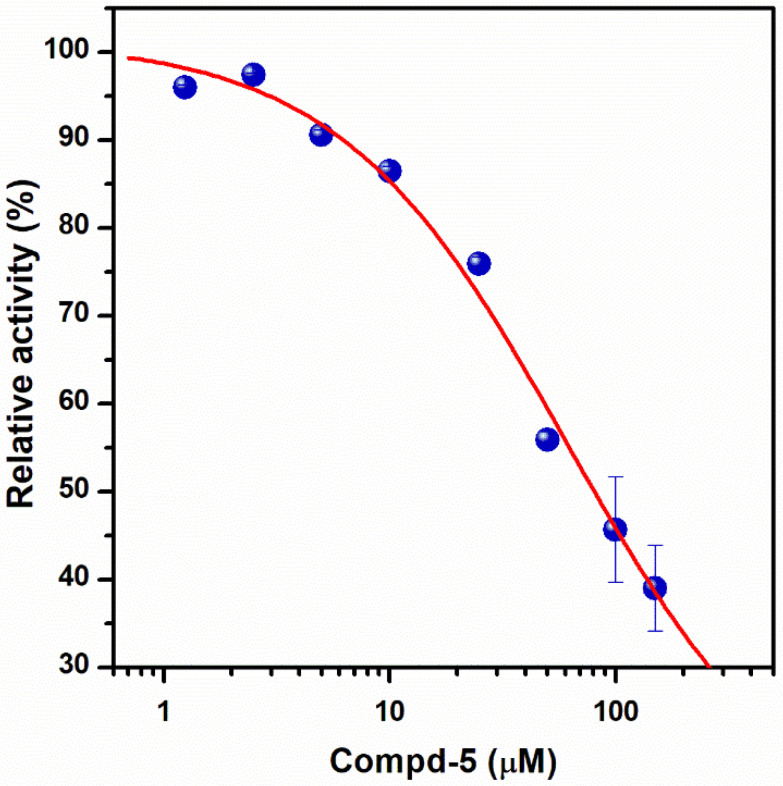
Dose-response plot of the inhibitory effect of Compd-5 on EcF_1_. Residual ATPase activity was measured using compound concentrations in the 1.25–150 μM range, in a 100 mM Tris-SO_4_ buffer solution with 1% DMSO (pH 8.0), 25 °C. The Hill equation was fitted to the experimental data, obtaining *IC*_50_ = 62 ± 5 μM, *h* = 0.86 ± 0.02, *v_r_* = 9 ± 1%. Data shown represent the average ± standard deviation of 3 independent experiments.

To further characterize the inhibitory effect of Compd-5, the ligand that exhibited the most potent activity, dose-response measurements were performed (Figure 3). Nonlinear analysis of the data, using the Hill equation, yielded an *IC_50_* value of 62 ± 5 μM and a Hill coefficient of 0.86 ± 0.02, suggesting that there is no cooperativity between the three β subunits. The fitting showed a residual enzyme activity of 9 ± 1% under saturation conditions, showing that Compd-5 nearly acts as a dead-end inhibitor.

All the active compounds had an NH group, serving as a hydrogen donor to the G^378^ carbonyl moiety. As an example, the predicted binding pose of Compd-5 is shown in Figure 2C. It is worth mentioning that Compd-5, Compd-14 and Compd-19 all come from the same family, being the only compounds in the tested set with the 4-(6-phenylpyridazin-3-yl)morpholine substructure (Simplified Molecular Input Line Entry System, SMILES, “[NH]c1cc(-c2nnc(N3CCOCC3)cc2)ccc1”). The binding energy (ΔG_PB_) of the positive inhibitors was computed on the corresponding docked poses, using the molecular mechanics Poisson–Boltzmann surface area (MM-PBSA) method [49,50] and compared against the rDock scores (ΔG_rDock_) in Table 1. Clearly, there is a better correlation of the degree of experimental inhibition with the energies calculated with rDock (*r*^2^ = 0.77) than with the MM-PBSA method (*r*^2^ = 0.12), which highlights the good performance of the HTVS method [43]. Finally, we computationally explored the possibility that the engineered inhibitors could also bind to the HTH motif of the other two β subunits. The pose of Compd-5 in β_1_ was used to dock the ligand on the other two subunits to perform unrestrained MD simulations. Like β_1_, β_3_ kept the inhibitor bound for up to 50 ns in two replicas, a suitable time to consider the interaction as stable [51]. In contrast, the compound was consistently released from β_2_ within the first nanoseconds of simulation (Figure S1).

### 2.2. De Novo Design of Peptide Inhibitors of EcF_1_ Targeting the βCterm

**Figure 4:**
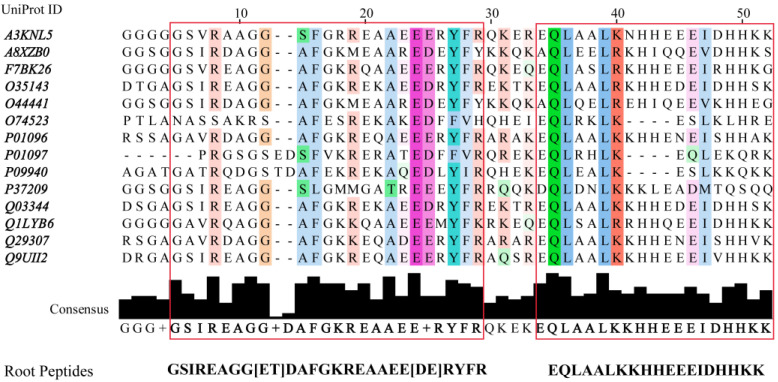
Multiple Sequence Alignment (MSA) performed with Multiple Alignment using Fast Fourier Transform, MAFFT, [55] for IF1 inhibitors deposited in the UniProt database. Consensus regions are identified at >40% of identity threshold at each position of the MSA. The resulting consensus regions are framed in a rectangular box. From them two root peptides were estimated IF1 sources: A3RKNL5, zebrafish. A8XZB0, Caenorhabditis briggsae. F7BK26, western clawed frog. O35143, mouse. O44441, Caenorhabditis elegans. 074523, fission yeast. P01096, bovine. P01097, baker’s yeast. P09940, torula yeast. P37209, Caenorhabditis elegans. Q03344, rat. Q1LYB06, zebrafish. Q29307, pig. Q9UII2, human. Jalview ver: 2.11.1.4 was used to visualize the MSA and determine the consensus.

In a previous report, we introduced a de novo design method of EcF_1_ peptide inhibitors [46]. The new inhibitors were designed in silico from the interfaces connecting F_O_F_1_-ATP synthase subunits, thus, proving the suitability of these scaffolds for the generation of a new family of inhibitors. Peptide libraries were built by applying simulated molecular evolution approaches, represented by the ROSE (random model of sequence evolution) algorithm [52], and later screened using PPI-Detect, a protein–protein interaction predictor [53], to score the binding likelihood of the peptides and EcF_1_. This new in silico strategy, guided by evolutionary and machine-learning-based methods, allowed widening and exploring the relevant structural space from natural peptide fragments to generate novel protein binders [46]. Here, we leveraged this approach for the de novo design of EcF_1_ peptide inhibitors, specifically targeting the βCterm. The fourteen IF1 sequences registered in the UniProt database [54], ranging from 42 to 50 aa length, were aligned to identify conserved regions. Two conserved regions were identified in the multiple sequence alignment (MSA), resulting in two consensus regions that were considered root or parent peptides (Figure 4).

ROSE operates by introducing stochastic point mutations into the root amino acid sequence, which is guided by a binary phylogenetic tree and a mutability vector, representing the conservation degree of each position in the sequence. Both the root peptide and the mutability vector are obtained by multiple sequence alignment of the selected set of peptides, carried out with Multiple Alignment, using Fast Fourier Transform, MAFFT, [55] (Table S2). The obtained library was then screened using PPI-Detect, which classifies and ranks the peptides as putative binders of the targeted site [46,53]. This strategy allowed us the rational exploration of the sequence space around the selected templates. From the root peptides, 385 unique mutants were generated. These peptides have a minimum identity, relative to their root sequence, of 70 %. Using PPI-Detect [53], these candidates were screened based on their interaction likelihood with the subunit β of EcF_1_ and the human sector (HsF_1_). Selected candidates had to meet the following criteria: (a) Peptides with maximum interaction likelihood with HsF_1_ below 0.5. (b) Peptides with maximum interaction likelihood with EcF_1_ above 0.5. (c) The difference between the interaction probabilities with *E. coli* and human enzymes is at least 0.1. After applying these selection criteria, three peptides were filtered out (Table 2). Surprisingly, the selected peptides showed the highest interaction score and score difference with the central domain of the β subunit (‘smart00382’ domain), while a score value of ~0.32 was obtained for the HTH motifs of both EcF_1_ and HsF_1_.

**Figure 5:**
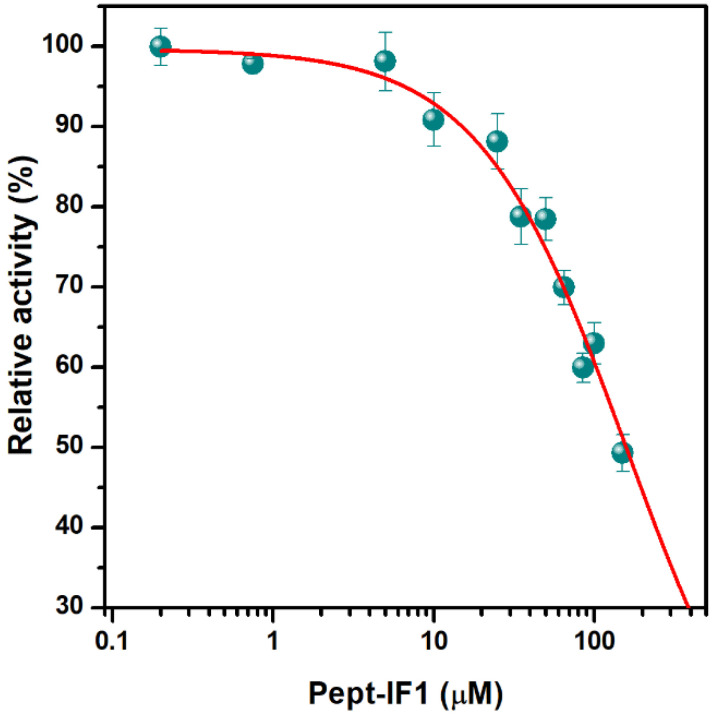
Dose-response plot of the inhibitory effect of Pept-IF1 on EcF_1_. Residual ATPase activity was measured using peptide concentration in the 1.25–150 μM range, in a 100 mM Tris-SO_4_ buffer solution with 1% DMSO (pH 8.0), 25 °C. The Hill equation was fitted to the experimental data, obtaining *IC*_50_ = 155 ± 14 μM, *h* = 1.1 ± 0.1, *v_r_* = −1 ± 3%. Data represent the average ± standard deviation of 3 independent experiments.

The negative GRAVY index evidences the polar features of these peptides and their potentially good solubility. From them, the candidate termed as Pept1-IF1 was selected for synthesis because of its neutral charge and isoelectric point value, close to 7.0. This peptide (sequence: Ac-GSIREAGGTHAFGKRESAEEERYFR-NH_2_) showed inhibitory activity against EcF_1_, with *IC_50_* = 155 ± 14 μM, *h* = 1.1 ± 0.1, and *v_r_* = −1 ± 3% (Figure 5).

### 2.3. Sequence Conservation of the HTH Motif in Bacteria

**Figure 6:**
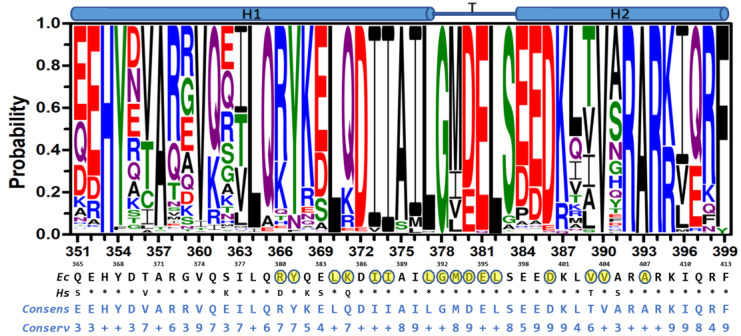
Conservation of the HTH motif in bacteria. Residue numbering in the up and down rows corresponds to the *E. coli* and human sequences, respectively. Multiple sequence alignment of 23,125 entries was performed with Clustal Omega [59]. Logos were generated using the Weblogo3 server [58]. Consensus, *E. coli* (*Ec*) and human (*Hs*) sequences are shown in the x-axis for comparison. Human residues identical to *E. coli* residues are shown with asterisks. The *Conserv* row corresponds to a conservation scale ranging from 0 (null conservation) to 10 (= +, complete conservation of physicochemical properties of the amino acid group) as defined in [62]. Residues within 5 Å of Compd-5 are highlighted in yellow.

Given the observed inhibition results and the functional relevance of the HTH motif, we set out to explore the sequence conservation of this motif in bacterial species. From the UniProt database, 23,125 b-subunit sequences, from all bacterial F_O_F_1_-ATP synthases, were retrieved [54]. The dataset was aligned to generate sequence logos of the HTH motif [58,59]. As previously observed in less comprehensive sequence analysis [60,61], the HTH motif is significantly conserved among bacteria (Figure 6), and even more conserved among Mammalia (Figure S2). The HTH motif comprises the α-helix 1 (H1: R^351^-I^376^), the turn (T: L^377^-S^383^), and the α-helix 2 (H2: E^384^-R^399^) segments (*E. coli* numbering). The ^380^DELSEED^386^ motif is within the C-terminal and N-terminal regions of T and H2, respectively. The most conserved regions encompass the central region D^370^-K^387^ (or HTH tip, which besides the DELSEED, also includes the largely conserved ^372^DIIAILG^378^ segment), the C-terminal segment ^392^RARKI^396^, plus some scattered, mostly hydrophobic residues, in H1 and H2. An analysis of the experimental 3D structures of F_1_ reveals that, with few exceptions, the most conserved residues in the HTH motif also form interactions with highly conserved residues, located either in the same subunit (mostly in the HTH motif itself) or in the adjacent α, β, or γ subunits (Figure S3). Further, 18 out of 45 residues of the HTH motif show significant variability. The most variable segment is in H1, while the most conserved is in T. Importantly, the binding site of the compounds, designed herein, includes portions of the conserved DIIAIL and DELSEED segments, as well as some moderately conserved residues. In particular, the Bacilli class shows the most contrasting differences regarding the human enzyme (Figure S4), a characteristic that could be exploitable to optimize molecules capable of selectively recognizing pathogens of this taxonomic group, and that do not bind to the human enzyme.

## 3. Discussion

Although the declining trend of newly approved antibiotics has recently reversed, infections caused by AMR bacteria are still an alarming threat to global public health [4]. Interfering bioenergetic pathways is an emerging strategy to combat pathogens [6]. Indeed, pharmacological-approved bedaquiline has attested that ATP synthase inhibition can be successfully harnessed to target aerobic organisms, such as *M. tuberculosis* [63]. In addition, AMR facultative anaerobes, including the ESKAPEE pathogens *S. aureus* and *E. coli*, lose resistance towards antibiotics upon ATP synthase inhibition [64,65]. Given the major role played by βCterm in inter-subunit communication, orchestrating the rotary mechanism of ATP synthase, we strived to design molecules capable of selectively targeting the HTH motif. Inspired by the effect that endogenous regulatory subunits [66,67,68], and some peptide venoms [69] and other exogenous inhibitors [26] have on the F_1_ subcomplex, the underlying idea was that by interfering with the conformational changes that the HTH motif undergoes, an inhibitory effect on the enzymatic activity can be achieved. To do this, we used two widely different in silico design strategies, one based on target 3D structure [70] and the other on peptide sequence data mining [46], obtaining organic molecules and an IF1-derived peptide, whose inhibitory potencies against EcF_1_ were in the micromolar range, comparable to those of known natural inhibitors, such as polyphenols and venom peptides, among others [28,29,71].

It has been shown, for an increasing number of proteins, that solvent site clusters map both orthosteric and allosteric sites [43,72]. Thus, besides identifying critical interaction points with substrates or natural ligands, MDmix-determined solvent sites have proved valuable as pharmacophoric restraints in HTVS [44,73], improving the rate of true-positive hits and the discovery of new kinds of inhibitors and binding probes [74,75]. Furthermore, by relying exclusively on the interactions determined by the force field and the kinetic energy of the atoms in the system, MDmix unbiasedly maps entire protein surfaces, opening a window of opportunity to identify potential allosteric sites that may be difficult to detect experimentally or by knowledge-based potential methods [76,77]. In this work, MD-determined solvent sites were used to guide the docking of drug-like molecules on the HTH motif. The best-ranked hits obtained from our HTVS were further filtered by steering MD, a technique that has been used to develop new kinds of inhibitors against HSP90 [45] and several oncogenic tyrosine-kinases [78]. The combined use of these orthogonal approaches that evaluate equilibrium- and trajectory-derived energies, respectively, allowed us to identify and experimentally validate novel inhibitors of EcF_1_.

The five inhibitory compounds (Table 1) showed a nitrogen atom that hydrogen bonded to, the carbonyl oxygen of the completely conserved G^378^ at the beginning of the HTH turn. In addition, hydrophobic contacts were established with H1 and H2 residues. Taken together, Compd-5, Compd-14 and Compd-19, the three 4-(6-phenylpyridazin-3-yl)morpholine-containing compounds, suggest the amide/sulfonamide position is amenable to a broad range of substituents and could be used to increase potency and modulate physicochemical properties. It is worth mentioning that Compd-5, our most potent inhibitor, is a relatively small molecule (MW = 378.2 Da), providing the opportunity to add chemical groups to it, to obtain more potent molecules. To our knowledge, no other activity for this compound has been reported so far [79]. Compd-7, our second-best inhibitor, with an overall different chemical structure, has a dimethylmorpholine moiety and, like Compd-14, a methoxyphenyl group. Compd-19 has no morpholine moiety, and the nitrogen with which it would hydrogen bond to, G^378^, is within a hydroxypyrimidine. To our knowledge, these are the first reported inhibitors designed to bind to a site formed within the HTH motif of the ATP-synthase β subunit.

In a previous report, we introduced a de novo design method of EcF_1_ peptide inhibitors [46]. The new inhibitors were designed in silico from the interfaces connecting F_O_F_1_-ATP synthase subunits, through a combination of simulated molecular evolution [52] and protein–protein interaction prediction [53] algorithms. The in vitro inhibitory capacity of the designed peptides proved the suitability of these scaffolds and the strategy for the generation of new inhibitor families. In this work, we derived new peptide sequences from known IF1 sequences. In contrast to the root IF1 peptides, which are incapable of inhibition of bacterial F_1_ ATPase [24,80], Pept-IF1 inhibited EcF_1_ with micromolar potency. However, to verify whether Pept-IF1 exhibits species discrimination and to determine its actual binding site on the β subunit, further experimental characterization is needed.

Antibiotics require tuned selectivity to achieve reliable discrimination between the pathogen target and the human or animal ortholog. Bedaquiline was initially proposed as a specific antibiotic for some species of the *Mycobacterium* genus. However, recent evidence has shown that the human enzyme is also susceptible to this antibiotic [81]. In addition, the *c* subunit, the binding target of bedaquiline, shows a low conservation among bacterial ATP synthases (e.g., mean identity of 33 ± 9 vs. 62 ± 8% of β subunits). Thus, unsurprisingly, bacteria can also evade this antibiotic, through mutations in the *c*-subunit-encoding *uncE* gene [82]. In contrast to the bedaquiline-binding site (mean identity 39 ± 20% in all bacteria), the HTH motif encompasses highly conserved sequence segments intercalated with variable positions (mean identity 69 ± 10% among bacteria). Furthermore, many of the HTH-conserved residues establish inter/intracatenary contacts with other highly conserved residues (Figure S3). Therefore, the HTH motif may offer a suitable target for allosteric drug discovery [83], as recently epitomized by the design of allosteric inhibitors against bacterial and viral enzymes, using conserved residues as binding anchors [84,85].

Site-directed mutagenic studies have unveiled that the rotary mechanism withstands severe changes in the HTH sequence [20,61,86,87,88,89,90,91,92]. Indeed, this rotational robustness is rooted in the fact that the γ-less α_3_β_3_ subcomplex, although in a largely decreased way, exhibits catalysis and alternating conformational changes [93], while isolated α and β subunits also undergo nucleotide-induced rearrangements that resemble those observed in the F_1_ subcomplex [21,94]. *Bacillus* PS3 enzymes, with deletions of up to 9 or 13 residues in HTH, keep catalytic activity in the synthesis or hydrolysis direction, respectively [20,61,90]. These results, together with point mutations in the HTH tip, led to the proposal that “…the physical length, rather than residue-specific interactions, of helix-1 is important for torque generation” [20], while the high conservation of some residues is due to the interaction that they establish with the regulatory subunits of the enzyme [88]. However, it has been repeatedly observed that in vitro mutations of the HTH sequence led to modifications of highly variable magnitude in the catalytic activity of the enzyme. The effects of these perturbations on oxidative phosphorylation and other ATP synthase-coupled processes on metabolic homeostasis have been scarcely studied [95,96]. Although it remains to be validated whether the inhibition of ATP synthase through molecules that bind the HTH motif is a feasible route for the development of new antibiotics, the most relevant finding of our study is the possibility of computationally predicting and validating novel sites of allosteric modulation, as biological evolution has repeatedly proved, with multiple sites for endogenous and exogenous inhibitors of this enzyme. This opens the door to the search for new pharmacological strategies, not only to attack infectious agents, but also to develop ATP synthase pharmacological modulators in metabolic and cellular contexts, where this enzyme plays a relevant role in the progression and establishment of pathologies [97,98].

## 4. Materials and Methods

### 4.1. Molecular Dynamics Simulations

MD trajectories were performed with the AMBER 14 suite using the FF99SB force field [99]. All simulations were carried out using the crystal structure of EcF_1_ (PDB ID 3oaa [22]). Modeling of protein missing atoms, N- and C-termini capping, and protonation at pH 7.4 were carried out with the Molecular Operating Environment (MOE, [100]). Using AMBER’s tLeap, the protein was placed in a truncated octahedral box spanning 18.0 Å further from the solute in each direction and solvated using a preequilibrated box of solvent containing pure water or 20% *v*/*v* ethanol/water. TIP3P water model was used. The system was first geometrically optimized (5000 cycles) to adjust the solvent orientation and eliminate local clashes, using the steepest descent algorithm. Initial velocities were assigned to get a 150 K distribution. The temperature was slowly raised to 300 K in 0.8 ns keeping the volume constant. The system was further equilibrated for one ns at 300 K in the NPT ensemble. The production was run in the NPT ensemble, using periodic boundary conditions. Temperature and pressure control were achieved using the Langevin thermostat and Berendsen barostat, respectively. Long-range electrostatic interactions were accounted for using the particle-mesh Ewald summation method as implemented in the PMEMD module of the AMBER suite, with a cut-off value of 9.0 Å to split direct electrostatics and Ewald summation [101,102]. The SHAKE algorithm was enabled and the integration timestep was 2fs. Running scripts were set up with the help of the pyMDMix software [43,103]. Trajectory analysis was performed with CPPTRAJ [104] and Chimera UCSF v14.1 [105]. Trajectories were run in triplicate. All the structure drawings were generated with ChimeraX [106].

### 4.2. Identification of Solvent Sites, Guided Docking, and Dynamic Undocking

Solvent sites were determined using the MDmix method as described elsewhere [43]. After trajectories were aligned, density maps for probe atoms were obtained by building a static mesh of grids over the entire simulation box and counting appearance of probe atoms in each grid during the trajectory. The observed appearance was converted into binding free energy (ΔG_SS_) applying the Boltzmann relationship, considering the observed probe atom distribution with an expected distribution in bulk solvent at 1.0 M. Solvent sites were filtered imposing an energy threshold of –1 kcal/mol. Compound libraries from Specs, Asinex, Enamine, Vitas M, ChemBridge, Key Organics, Princeton Biomolecular Research, and Life Chemicals, with a total of ~8 × 10^6^ molecules, were docked using rDock [44]. Solvent sites were used as pharmacophores to filter compound libraries. A penalty score that increased proportionally to the square of the distance to the required solvent sites was applied when the distance was larger than 2 Å. Best ranked ligands were further filtered using steered molecular dynamics (SMD) simulations using the dynamic undocking method DUck as described elsewhere [45]. A total of 100 SMD simulations yielding 50 ns per ligand were run, imposing harmonic restraints with a force constant of 1.0 kcal/molÅ^2^ on all receptor non-hydrogen atoms to preserve the protein conformation. Compounds were filtered out according to the work (ΔG_QB_) needed to separate the ligand’s atom forming a hydrogen bond with the β-subunit G^378^ carbonyl oxygen to a 5.0 Å distance, using a cutoff value of 6.0 kcal/mol. A workflow illustrating the process from the identification of solvent sites to dynamic undocking has been published elsewhere [78].

### 4.3. Engineering Peptide Inhibitors via Evolutionary and Protein–Protein Interaction Algorithms

**Figure 7:**
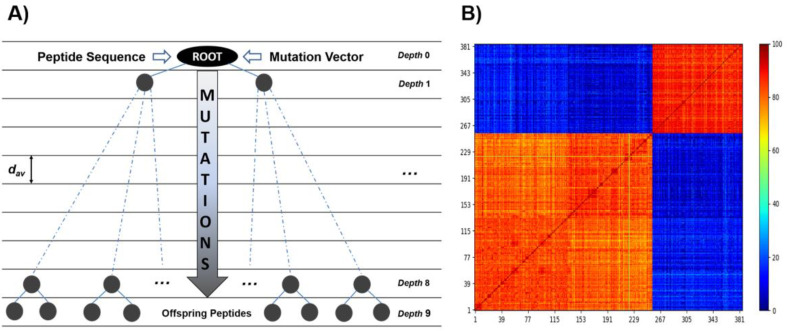
In silico design of a diversity-oriented peptide library. (**A**) Workflow illustrating the application of ROSE algorithm (https://bibiserv.cebitec.uni-bielefeld.de/rose, accessed on 12 March 2019). Root peptides and their corresponding mutation vectors are the input data. Besides the mutation vectors, ROSE also uses a binary tree to guide the stochastic point mutations on the root peptides. The binary tree topology is determined by the number of nodes (1023), depth (9) and average distance (dav = 5–20 PAMs). ROSE was calibrated to keep a minimum identity of ~70% of the generated peptides with the corresponding root sequence. The obtained library was composed of 385 unique peptides. (**B**) Heatmap showing the identity matrix among the generated peptides. Two blocks are distinguished, which corresponds to the root peptides selected from different fragments of IF1.

IF1-based peptide inhibitors were derived as recently described elsewhere [46]. IF1 sequences retrieved from the UniProt database [54] were used to estimate consensus sequences that served as the root peptide to generate offspring candidates (peptide library) by applying simulated molecular evolution approaches, represented by the ROSE (random model of sequence evolution) algorithm [52] (Figure 7). The structural diversity generated by ROSE is guided by evolutionary parameters, which were tuned to develop a diversity-oriented sampling around the root sequence. The library was subsequently screened using PPI-Detect, a protein–protein interaction predictor [53,107], to score the binding likelihood of the peptides and EcF_1_.

### 4.4. Protein Production and Purification

Unless stated otherwise, all the chemicals were from FORMEDIUM (Norfolk, UK). EcF_1_ was recombinantly expressed in *E. coli* strain DK8 using the pBWU13.4 plasmid containing the *unc* operon [108]. Briefly, *E. coli* membranes carrying EcF_1_ were first washed in the presence of protease inhibitors 6-aminohexanoic acid and p-aminobenzamidine, and finally in the presence of the former to solubilize the enzyme. The subcomplex was then purified by ion exchange and size exclusion chromatography using Whatman DE52 Cellulose and Sephacryl S-300 resin columns. Protein concentrations were determined using the Pierce BCA Protein Assay Kit (Thermo-Fisher, Waltham, MA, USA).

### 4.5. ATPase Activity Assays

The malachite green assay was used to determine ATPase activities as previously described [109]. All compounds were assayed in a concentration range spanning 0.05–150 µM, incubated with 10 nM (0.5 pmol) *Ec*F_1_ (50 mM Tris-SO_4_ pH 8.0, 1% DMSO) for 1 h at 30 °C in a total volume of 30 µL in 96-well microplates. Reactions were started by adding 20 µL 1 mM MgATP, incubated at 25 °C for 2 min and then stopped with 200 µL of 3.28 M H_2_SO_4_ and 15 mM (NH_4_)Mo_7_O_24_ solution. Absorbance was read at 610 nm using a microplate reader (Biotek). The ATP regenerating system [110] was also used to determine ATPase activities. Experiments were carried out in a 50 mM KCl, 3 mM MgCl_2_, 1.5 mM phosphoenolpyruvate, 300 mM NADH, 50 mM Tris pH 8.0, buffered solution incubating 3 U of rabbit pyruvate kinase (Merck Inc., Kenilworth, NJ, USA), 4.2 U of rabbit lactic dehydrogenase (Merck Inc.), 5.2 nM EcF_1_ with 100 µM of assayed compounds, for 1 h at 30 °C in 120 mL per well in 96-well microplates. Reactions were started by adding 30 mL of a 50 mM KCl, 3 mM MgCl_2_, 1.5 mM phosphoenolpyruvate, 300 mM NADH, 50 mM Tris pH 8.0 solution, including 1 mM ATP. ATPase activity was monitored through absorbance changes at 340 nm for 2 min in a microplate reader (Biotek, Winooski, VT, USA).

The concentration of inhibitor required to achieve a 50% reduction in enzymatic activity, *IC_50_*, was obtained using the Hill equation:

$$\frac{v_{i}}{v_{0}}=\frac{{\left[Inh\right]}^{h}}{IC_{50}{}^{h}+{\left[Inh\right]}^{h}}+v_{r}$$

where *v_0_* and *v_i_* are the initial catalytic velocities in absence and in presence of a given concentration of the inhibitor molecule, [*Inh*], *h* is the Hill coefficient and *v_r_* is the residual velocity under saturation conditions by the inhibitor.

### 4.6. Sequence Analysis of the HTH Motif

ATP synthase b subunits sequences from bacteria and Mammalia taxa were retrieved from the UniProt database [54]. Jalview2 [111] was used to curate the database, excluding redundant sequences (identity < 100%), yielding 23,125 and 142 sequences for bacteria and Mammalia groups, respectively. The curated database was used to generate a multiple sequence alignment with Clustal Omega [59]. Sequence logos of the HTH motif were generated using the Weblogo3 server [58]. Very similar logo results were obtained for both taxonomic groups using a redundancy sequence identity cutoff of <98% or <99%.

## Figures and Tables

**Table 1:** Summary of the final active compounds designed against the HTH motif structure of EcF_1_.

	Structure *^a^*	ΔG_rDock_ *^b^* (kcal/mol)	W_QB_ *^c^* (kcal/mol)	ΔG_PB_ *^d^* (kcal/mol)	Residual ATPase Activity (%) *^e^*	logS *^f^*
**Compd-5**	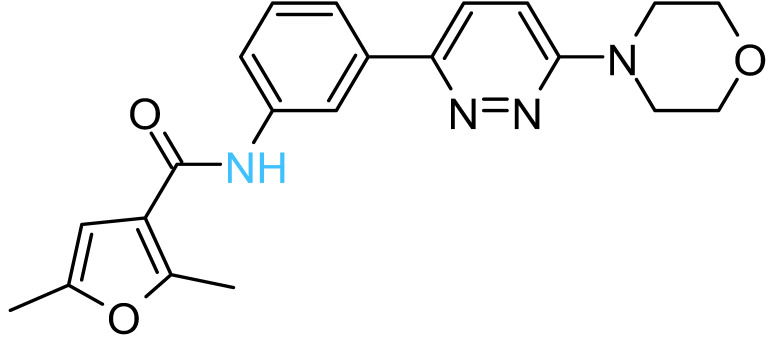	−6.0	8.7	−29	43 ± 6 (50 ± 5%)	−4.7
**Compd-7**	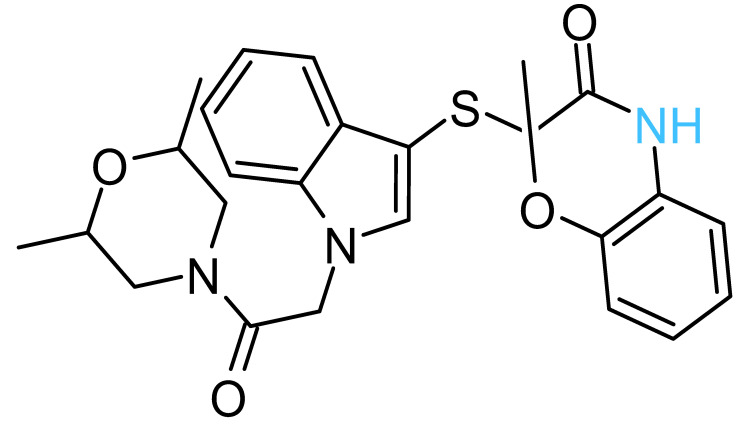	−5.5	6.0	−30	64 ± 12 (70 ± 10%)	−5.1
**Compd-14**	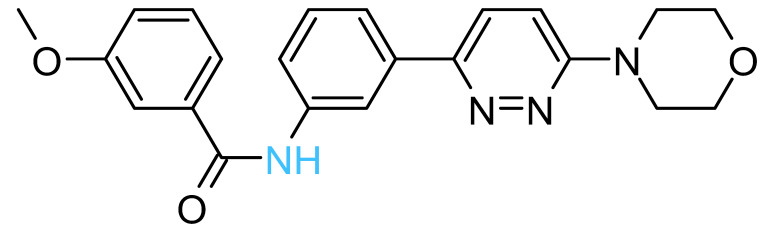	−4.5	6.7	−25	75 ± 8 (73 ± 2%)	−5.3
**Compd-15**	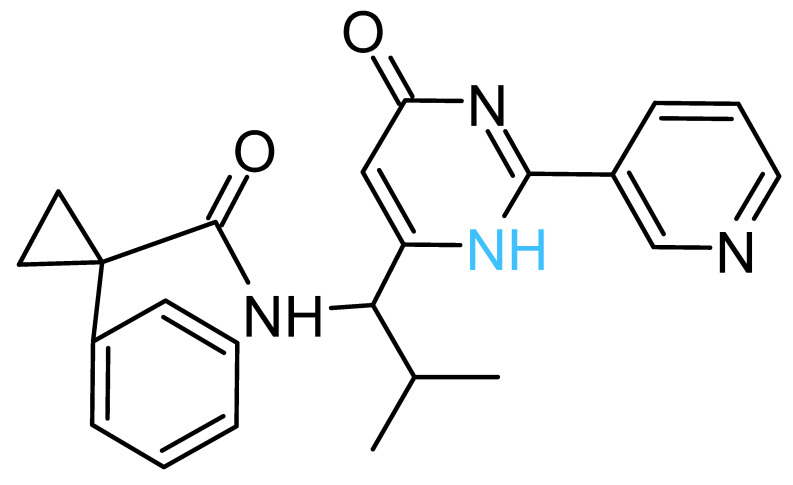	−4.3	6.0	−24	67 ± 5 (ND)	−4.9
**Compd-19**	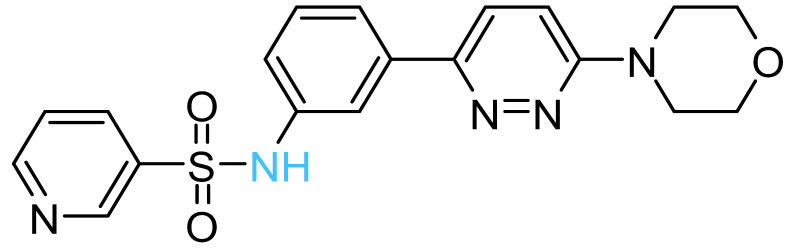	−4.0	6.0	−29	77 ± 7 (70 ± 10%)	−2.8

*^a^* NH atoms that established hydrogen bonds with the G^378^ backbone oxygen are in blue. *^b^* rDock score, a weighted sum of intermolecular, ligand intramolecular, site intramolecular and pharmacophoric restraints [44]. *^c^* Work needed to separate the ligand’s atom forming a hydrogen bond with the protein to a 5 Å distance, calculated with DUck [45]. *^d^* Molecular mechanics Poisson–Boltzmann surface area (MM-PBSA) calculated free energy [49,50]. *^e^* Residual ATPase activity of EcF_1_ determined by the malachite method (and the ATP regenerating system, values in parentheses), incubated with 100 µM of the indicated compound, in a 50 mM Tris-SO_4_ buffer solution with 1% DMSO (pH 8.0), 25 °C. Data represent the average ± standard deviation of at least 3 independent experiments. ND, not determined. *^f^* Predicted aqueous solubility determined with the Chemaxon solubility predictor server.

**Table 2:** Interaction scores and chemical–physical properties of the selected peptide candidates.

Peptide	Sequence	Score *^a^* (EcF_1_)	Score *^a^* (HsF_1_)	Charge (pI) *^b^*	GRAVY *^b^*
**Pept1-IF1**	GSIREAGGTHAFGKRESAEEERYFR	0.512	0.402	0 (6.78)	−1.292
**Pept2-IF1**	GSIREAGGTDGFGKREAAEEEKYGR	0.561	0.420	−1 (5.11)	−1.392
**Pept3-IF1**	GSVREAGGTGAFGKRESAEEERYFR	0.580	0.479	0 (6.34)	−1.192

*^a^* The domain ‘smart00382’ was mapped on the β subunits of EcF_1_ and HsF_1_ using the NCBI tool CD-Search [56] to identify conserved domains. The extracted fragments of the subunits were used to compute the interaction scores with the peptides. *^b^* Values calculated with ProtParam [57].

## Data Availability

Not applicable.

## Supplementary Materials

The following supporting information can be downloaded at: https://www.mdpi.com/article/10.3390/antibiotics11050557/s1, Table S1: Docked organic molecules on the HTH with best scores according to rDock and DUck methods; Table S2: Root peptides and mutation vectors for each peptide inhibitor family (IF1 and Heterogeneous Set); Figure S1: Distance between the carbonyl oxygen of G378 and the amino nitrogen of Compd-5 as a function of time. Figure S2: Conservation of the HTH motif in Mammalia class. Figure S3: Conserved contacts between residues of the HTH motif and neighboring subunits [112]. Figure S4: Conservation of the HTH motif in Bacilli class. [58,59,62,112] are cited in Supplementary Materials.
