# Supplementary material for: Computational Design of Inhibitors Targeting the Catalytic β Subunit of Escherichia coli FOF1-ATP Synthase
Source: Antibiotics (Basel). 2022 Apr 22;11(5):557. doi: 10.3390/antibiotics11050557 (PMC9138118; doi:10.3390/antibiotics11050557)
Supplement: Supplementary file 1 [file antibiotics-11-00557-s001.zip › antibiotics-1649425-supplementary.pdf]

Supplementary Information

Computational Design of Inhibitors Targeting the Catalytic β subunit of *Escherichia coli* F_OF_1-ATP synthase

Luis Pablo Avila-Barrientos [1,†], Luis Fernando Cofas-Vargas [1,†], Guillermin Agüero-Chapin [2,3], Enrique Hernández-García [1], Sergio Ruiz-Carmona [4,‡], Norma A. Valdez-Cruz [5], Mauricio Trujillo-Roldán [5], Joachim Weber [6], Yasser B. Ruiz-Blanco [1,7,*], Xavier Barril [8,9] and Enrique García-Hernández [1,*]

Universidad Nacional Autónoma de México, Instituto de Química, Ciudad Universitaria, Ciudad de México 04510, Mexico; lpablo@comunidad.unam.mx (L.P.A.-B.); fcofas@comunidad.unam.mx (L.F.C.-V.); enrique.hernandez@iquimica.unam.mx (E.H.-G.)

2 CIMAR/CIIMAR, Centro Interdisciplinar de Investigação Marinha e Ambiental, Universidade do Porto, Terminal de Cruzeiros do Porto de Leixões, Av. General Norton de Matos, s/n, 4450-208, Portugal; gchapin@ciimar.up.pt

3 Departamento de Biologia, Faculdade de Ciências, Universidade do Porto, Rua do Campo Alegre, 4169-007 Porto, Portugal

4 Institut de Biomedicina de la Universitat de Barcelona (IBUB) and Facultat de Farmàcia, Universitat de Barcelona, Av. Joan XXIII s/n, 08028 Barcelona, Spain; sruizcarmona@gmail.com

5 Programa de Investigación de Producción de Biomoléculas, Departamento de Biología Molecular y Biotecnología, Instituto de Investigaciones Biomédicas, Universidad Nacional Autónoma de México, Cd. Universitaria, 04510 Ciudad de México, Mexico; adri@biomedicas.unam.mx (N.A.V.-C.); maurotru@biomedicas.unam.mx (M.T.-R.)

6 Department of Chemistry and Biochemistry, Texas Tech University, Lubbock, TX 79409, USA; joachim.weber@ttu.edu

7 Center of Medical Biotechnology, Faculty of Biology, University of Duisburg-Essen, Essen, 45127, Germany

8 Departament de Farmacia i Tecnología Farmacèutica, i Fisicoquímica, Institut de Biomedicina (IBUB), Universitat de Barcelona, Av. Joan XXIII, 27-31, E-08028 Barcelona, Spain; xbarril@ub.edu

9 Catalan Institution for Research and Advanced Studies (ICREA), 08010 Barcelona, Spain

* Correspondence: yasser.ruizblanco@uni-due.de (Y.B.R.-B.); egarciah@unam.mx (E.G.-H.)

† These authors contributed equally to this work.

‡ Current address: Cambridge Baker Systems Genomics Initiative, Baker Heart and Diabetes Institute, Melbourne, VIC, 3004, Australia.

Table of content

**Table S1**	Docked organic molecules on the HTH with best scores according to rDock and DUck methods
**Table S2**	Root peptides and mutation vectors for each peptide inhibitor family (IF1 and Heterogeneous Set)
**Figure S1**	Distance between the carbonyl oxygen of G378 and the amino nitrogen of Compd-5 as a function of time.
**Figure S2**	Conservation of the HTH motif in Mammalia class
**Figure S3**	Conserved contacts between residues of the HTH motif and neighboring subunits
**Figure S4**	Conservation of the HTH motif in Bacilli class

Table S1. Docked organic molecules on the HTH with best scores according to rDock and DUck

Comp. ID	SMILES	Vendor	Vendor ID
1	Brc1cc(N2CC(CC2=O)C(=O)NCc2cc(NC(=O)c3cccnc3)ccc2)ccc1	Enamine	T6465774
2	s1cccc1CNC(=O)CSc1nc(nc2sc3CCCCc3c12)COc1ccccc1	Asinex	ASN_05445348
3	Clc1cc2c(N=C(NN=C2c2cc(OC)c(OCc3ccccc3)cc2)c2cccnc2)cc1	Asinex	ASN_4053622
4	O=C(NCc1cn(nc1-c1ccccc1)Cc1ccccc1)CCn1ncnc1	Enamine	T6020091
5	o1c(C)c(cc1C)C(=O)Nc1cc(ccc1)-c1nnc(N2CCOCC2)cc1	Life Chemicals	F2724_2447
6	Clc1cc(-n2c(nnc2SCC(=O)N2CCc3c2cccc3) CNC(=O)c2cc(OC)ccc2)ccc1	Life Chemicals	F0507_2237
7	S(CC(=O)Nc1ccccc1OC)c1c2c(n(c1)CC(=O)N1CC(OC(C1)C)C)cccc2	Life Chemicals	F2016_612
8	O=C(NC1CC2[NH+](C(C1)CCC2)Cc1ccc(cc1)C)c1cccnc1	Asinex	ASN_6224181
9	S(CC(=O)N1CCc2c1cccc2)c1nnc(n1-c1cc(OC)ccc1OC)CNC(=O)c1cc(OC)ccc1	Life Chemicals	F0772_947
10	s1cc(nc1C)CCNC(=O)CC(NC(=O)N)c1ccccc1C	Enamine	T6613814
11	O=C1N=C(NC(=C1)CNC(=O)c1n(nc(c1)C(C)(C)C)C)c1cccnc1	Asinex	SYN_19815273
12	Clc1cc(ccc1Cl)C[NH2+]C(C(C)C)C=1NC(=NC(=O)C=1)c1cccnc1	Asinex	SYN_19819116
13	S(CC(=O)NCc1ccccc1)c1c2c(n(c1)CC(=O)N1CCCCCC1)cccc2	Life Chemicals	F2016_444
14	O1CCN(CC1)c1nnc(cc1)-c1cc(NC(=O)c2cc(OC)ccc2)ccc1	Life Chemicals	F2724_260
15	O=C1N=C(NC(=C1)C(NC(=O)C1(CC1)c1ccccc1)C(C)C)c1cccnc1	Asinex	SYN_19818191
16	Ic1cc(NC(=O)Cn2c3c([nH+]c2NCC(O)C)cccc3)ccc1	Enamine	T6127320
17	Clc1ccc(Cl)cc1S(=O)(=O)Nc1cc(ccc1)C(=O)Nc1cc(OC)ccc1	Enamine	T5262939
18	Clc1ccc(cc1)-c1nn(cc1C1N(N=C(C1)c1ccccc1O)C(=O)C)-c1ccccc1	Enamine	T5806496
19	S(=O)(=O)(Nc1cc(ccc1)-c1nnc(N2CCOCC2)cc1)c1cccnc1	Life Chemicals	F2588_215
20	S(Cc1ccccc1)C(C(=O)N1CCC(NC(=O)c2cc(OC)ccc2)CC1)C	Enamine	T6604160
21	o1cc(cc1)C(=O)NC(C(=O)Nc1cc(ccc1)Cn1nccc1)C	Enamine	T6601933
22	O1c2cc(CNC(=O)c3cc4c(nc3)n[nH]c4C)c(OCC)cc2CC1C	Enamine	T6436978
23	O1CCC(CC1(C)C)(CCNC(=O)c1cnc(nc1C)C)c1ccc(OC)cc1	Asinex	SYN_15581643
24	s1cc(nc1NC(=O)c1cc2nc[nH]c2cc1)-c1ccc(cc1)CCC	Enamine	T5770195
25	s1cc(nc1NC(=O)c1cc2nn[nH]c2cc1)-c1ccc(cc1)CC	Enamine	T5957036
26	Oc1c(cccc1O)\C=N/c1cc2nc(n(c2cc1)C)CO	Enamine	T0502_6866
27	S(CC(=O)Nc1n(nc(c1)-c1cc(C)c(cc1C)C)-c1ccccc1)c1nncn1C	Enamine	T5237329

Table S2. Root peptides extracted from the MSA and their respective mutation vectors.

Inhibitor Class	***Root Peptide/Mutation Vector***
IF1	GSIREAGG[ET]DAFGKREAAEE[DE]RYFR
	[0.15, 0.40, 0.60, 0.10, 0.64, 0.20, 0.10, 0.2, 1.0, 0.9, 0.2, 0.1, 0.2, 0.2, 0.3, 0.2, 0.6, 0.1, 0.5, 0.0, 0.5, 0.5, 0.15, 0.2, 0.3]
IF1	EQLAALKKHHEEEIDHHKK
	[0.2, 0.0, 0.1, 0.5, 0.4, 0.0, 0.35, 0.4, 0.3, 0.4, 0.4, 0.4, 0.3, 0.4, 0.6, 0.3, 0.2, 0.6, 0.35]
Het. Inhibitors	RVYGVGNIILYEKA
Het. Inhibitors	SLKEIQEAIDLDRELAKLKQKP-PAIDWMYGKANMAKAPNV
Het. Inhibitors	TFEKPKFEALKVPIPEDLDYKYTAQVDAEEKE
Class-4	MENLNMDLLYMAAAVMMGLAAIGAAI-GIGILGGKFLEGAAR

Root peptides summarize structural information of the two most conserved regions within both inhibitor classes. Mutation vectors represent the root peptides by weighting each residue with its variability or conservation degree shown in the consensus fragments (Figure 5 in the manuscript). “Zero” value indicates no mutations (high conservation degree) while “one” value represents high mutation probability.

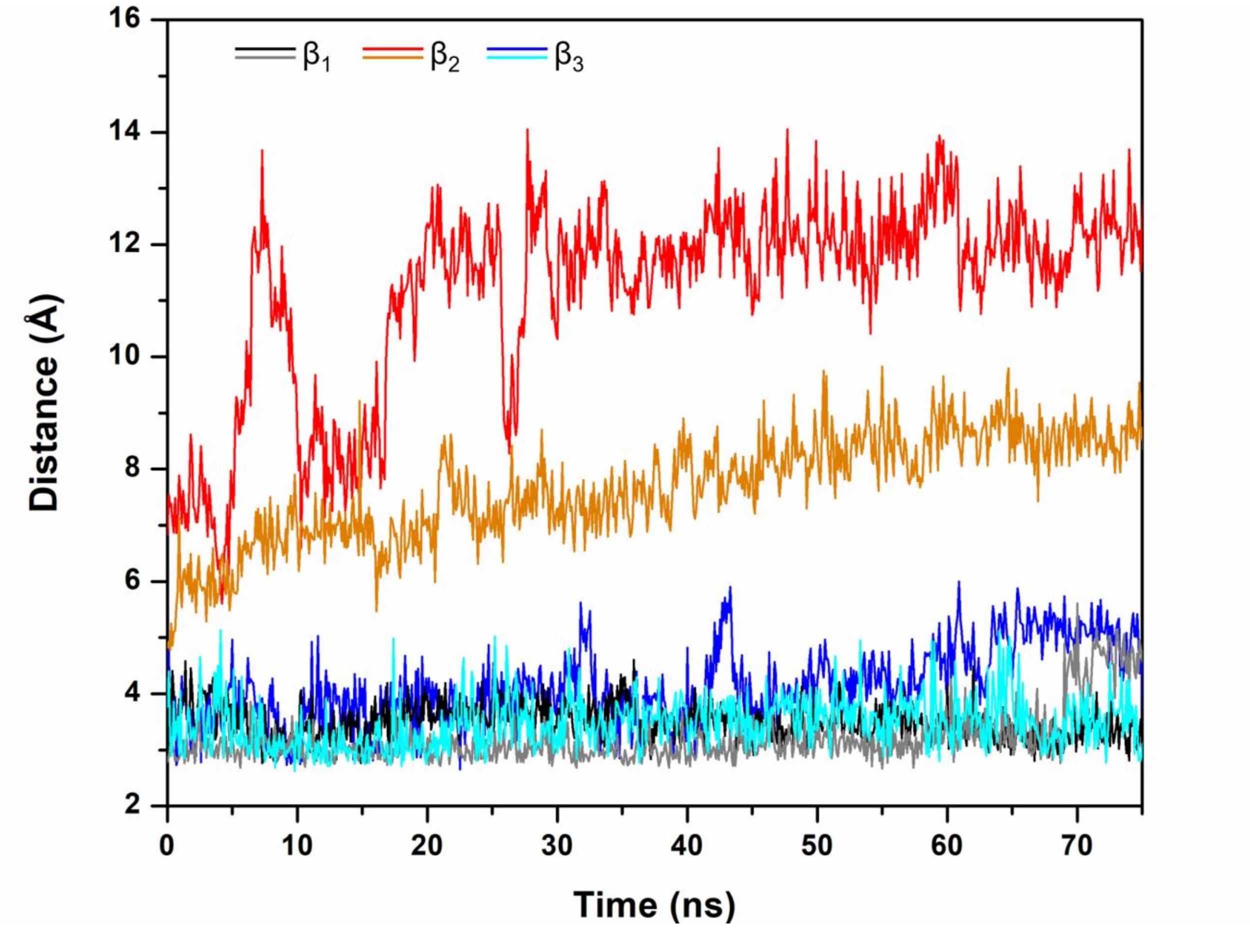

Figure S1. Distance between the carbonyl oxygen of G^{378} and the amino nitrogen of Compd-5 as a function of time. Two replicas of 75 ns were run with EcF_1 bound to three Compd-5 molecules. The Compd-5 pose in β_1 obtained by HTVS was transferred to β_2 and β_3. Two MD simulations were carried out.

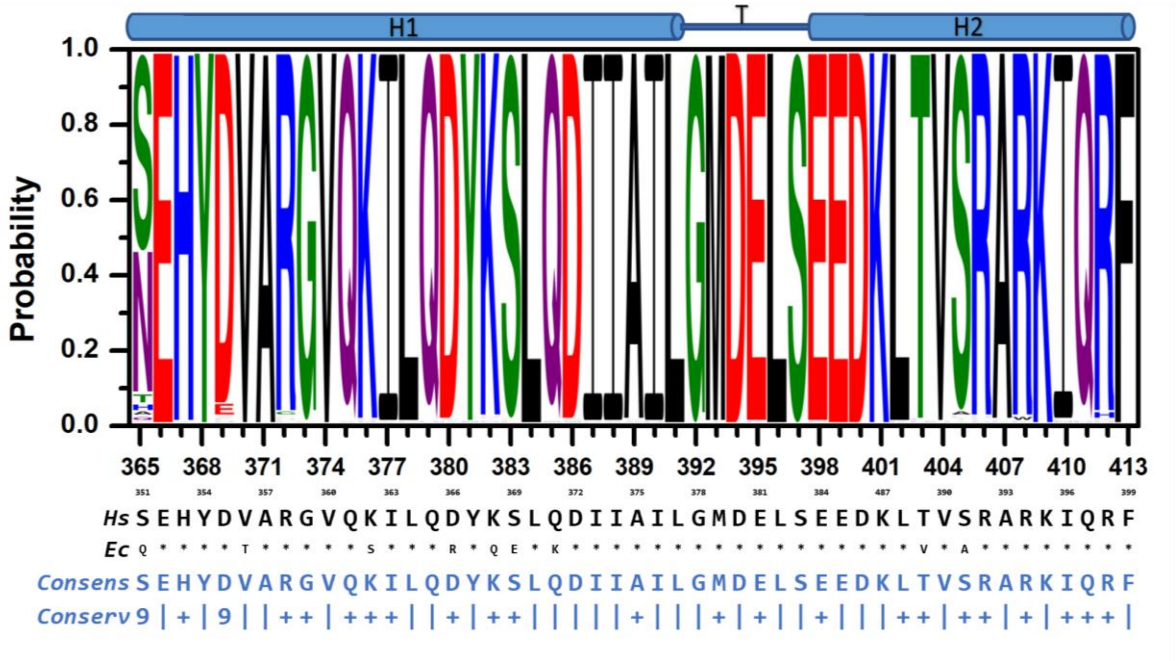

Figure S2. Conservation of the HTH motif in the Mammalia class. Residue numbering in the up and down rows corresponds to the human and *E. coli* sequences, respectively. Multiple sequence alignment of 142 entries was performed with Clustal Omega [1]. Logos were generated using the Weblogo3 server [2]. Consensus, human (*Hs*) and *E. coli* (*Ec*) sequences are shown in the x-axis for comparison. *E. coli* residues identical to human residues are shown with asterisks. The *Conserv* row corresponds to a conservation scale ranging from 0 (null conservation) to 10 (=+, complete conservation of physicochemical properties of the amino acid group) as defined in [3]. Absolute residue conservation is indicated by the symbol |.

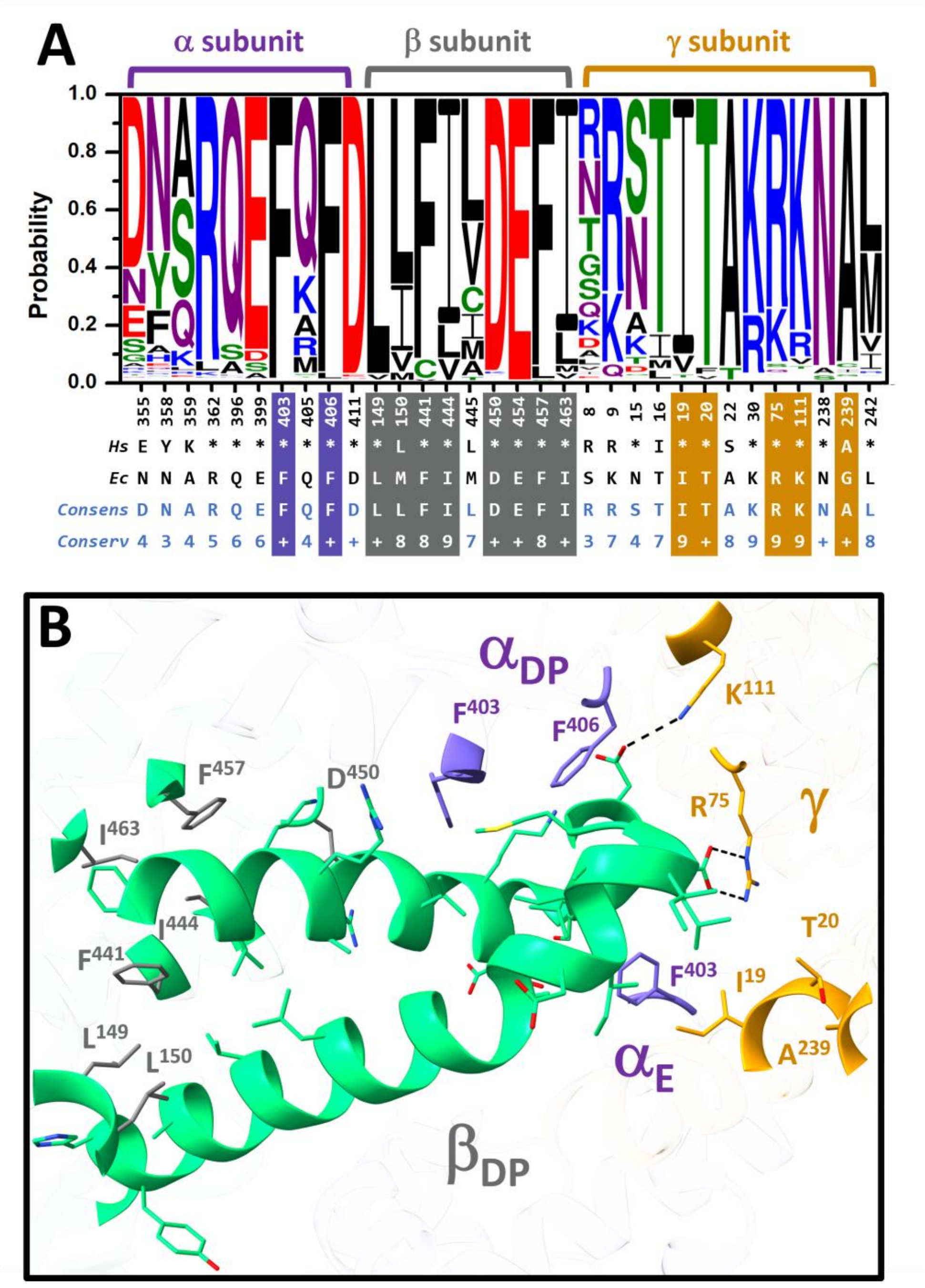

Figure S3. Conserved contacts between residues of the HTH motif and neighboring subunits in bacteria. The contacts were determined from the crystallographic structure of BsF1 in the so-called "ground state (pdb 2jdi, [4]). **A.** Logo graphic showing the residues of the α (purple), β (gray) and γ (ocher) subunits that contact the HTH motif. The residue numbering corresponds to that of the human/bovine enzyme. Highly conserved residues are highlighted. **B.** Schematic representation of conserved HTH residues (green) contacting conserved residues of neighboring subunits. The intrachain contacts of the β subunit are shown in gray.

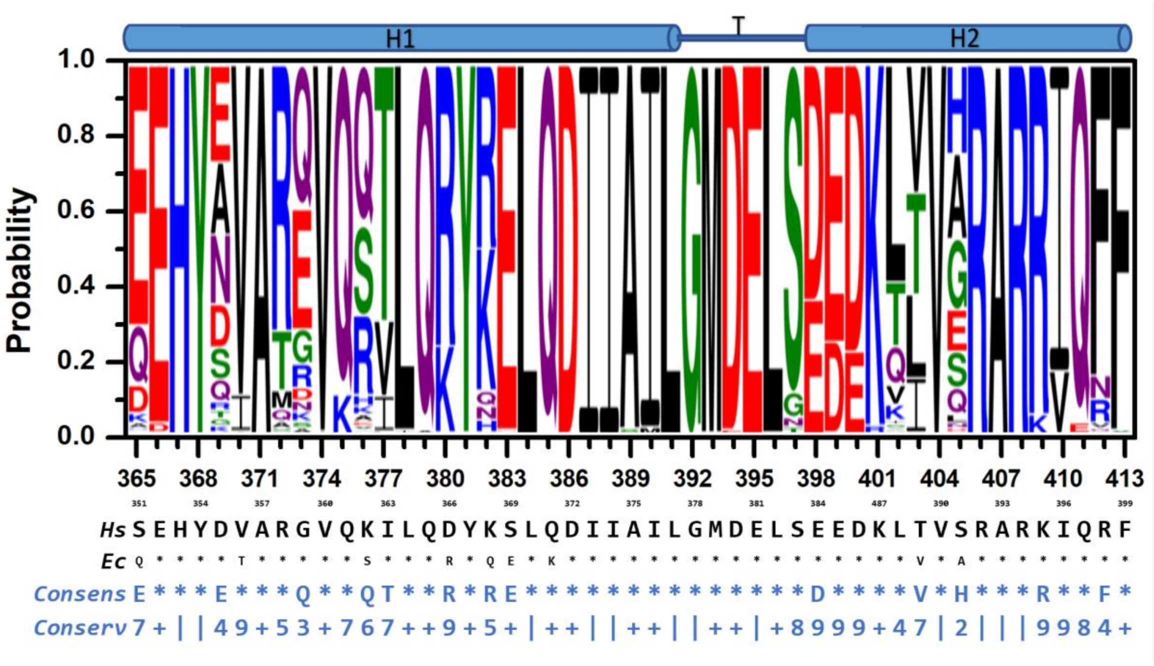

Figure S4. Conservation of the HTH motif in the Bacilli class. Residue numbering in the up and down rows corresponds to the human and *E. coli* sequences, respectively. Multiple sequence alignment of 444 entries was performed with Clustal Omega [1]. Logos were generated using the Weblogo3 server [2]. Bacilli consensus, *E. coli* (*Ec*) and human (*Hs*) sequences are shown in the x-axis for comparison. *E. coli* residues identical to human residues are shown with asterisks. The *Conserv* row corresponds to a conservation scale ranging from 0 (null conservation) to 10 (=+, complete conservation of physicochemical properties of the amino acid group) as defined in [3]. Absolute residue conservation is indicated by the symbol |.

References

1. Sievers, F.; Wilm, A.; Dineen, D.; Gibson, T.J.; Karplus, K.; Li, W.; Lopez, R.; McWilliam, H.; Remmert, M.; Söding, J.; et al. Fast, scalable generation of high-quality protein multiple sequence alignments using Clustal Omega. *Mol. Syst. Biol.* **2011**, *7*, 539. https://doi.org/10.1038/msb.2011.75.
2. Crooks, G.E.; Hon, G.; Chandonia, J.-M.; Brenner, S.E. WebLogo: A Sequence Logo Generator. *Genome Res.* **2004**, *14*, 1188–1190. https://doi.org/10.1101/gr.849004
3. Livingstone, C.D.; Barton, G.J. Protein sequence alignments: A strategy for the hierarchical analysis of residue conservation. Comput. Appl. Biosci. 1993, 9, 745–756. https://doi.org/10.1093/bioinformatics/9.6.745.
4. Bowler, M.W.; Montgomery, M.G.; Leslie, A.G.W.; Walker, J.E. Ground state structure of F_1-ATPase from bovine heart mitochondria at 1.9 Å resolution. *J. Biol. Chem.* **2007**, *282*, 14238–14242. https://doi.org/10.1074/jbc.M700203200.
